# Second-line chemotherapy after early disease progression during first-line chemotherapy containing bevacizumab for patients with metastatic colorectal cancer

**DOI:** 10.1186/s12885-021-08890-6

**Published:** 2021-10-29

**Authors:** Shun Yamamoto, Kengo Nagashima, Takeshi Kawakami, Seiichiro Mitani, Masato Komoda, Yasushi Tsuji, Naoki Izawa, Kentaro Kawakami, Yoshiyuki Yamamoto, Akitaka Makiyama, Kentaro Yamazaki, Toshiki Masuishi, Taito Esaki, Takako Eguchi Nakajima, Hiroyuki Okuda, Toshikazu Moriwaki, Narikazu Boku

**Affiliations:** 1 Department of Gastrointestinal Medical Oncology, National Cancer Center Hospital, Tsukiji 5-1-1, Chuo-ku, Tokyo, 1040045 Japan; 2 Department of Head and Neck, Esophageal Medical Oncology, National Cancer Center Hospital, Tsukiji 5-1-1, Chuo-ku, Tokyo, 1040045 Japan; 3 Research Center for Medical and Health Data Science, the Institute of Statistical Mathematics, Tokyo, 1908562 Japan; 4 Division of Gastrointestinal Oncology, Shizuoka Cancer Center, Shizuoka, 4118777 Japan; 5 Department of Clinical Oncology, Aichi Cancer Center Hospital, Aichi, 4648681 Japan; 6 Department of Gastrointestinal and Medical Oncology, National Hospital Organization Kyushu Cancer Center, Fukuoka, 8111395 Japan; 7 Department of Medical Oncology, Tonan Hospital, Hokkaido, 0600004 Japan; 8 Department of Clinical Oncology, St. Marianna University School of Medical Hospital, Kanagawa, 2168511 Japan; 9 Department of Medical Oncology, Keiyukai Sapporo Hospital, Hokkaido, 0030027 Japan; 10 Division of Gastroenterology, Faculty of Medicine, University of Tsukuba, Ibaraki, 3058575 Japan; 11 Department of Hematology/Oncology, Japan Community Healthcare Organization Kyushu Hospital, Fukuoka, 8068501 Japan; 12 Department of Medical Oncology and General Medicine, IMS Hospital, Institute of Medical Science, University of Tokyo, Tokyo, 1088639 Japan

**Keywords:** Metastatic colorectal cancer, Bevacizumab continuation beyond progression, Early disease progression, Second-line chemotherapy

## Abstract

**Background:**

The ML18174 study, which showed benefits of bevacizumab (BEV) continuation beyond progression (BBP) for metastatic colorectal cancer (mCRC), excluded patients with first-line progression-free survival (PFS) shorter than 3 months. The present study was conducted to evaluate the efficacy of second-line chemotherapy after early disease progression during first-line chemotherapy containing bevacizumab.

**Methods:**

The subjects of this study were mCRC patients who experienced disease progression < 100 days from commencement of first-line chemotherapy containing BEV initiated between Apr 2007 and Dec 2016. Second-line chemotherapy regimens were classified into two groups with and without BEV/other anti-angiogenic agents (BBP and non-BBP) and efficacy and safety were compared using univariate and multivariate analysis.

**Results:**

Sixty-one patients were identified as subjects of this study. Baseline characteristics were numerically different between BBP (*n* = 37) and non-BBP (*n* = 25) groups, such as performance status (0–1/> 2/unknown: 89/8/3 and 56/40/4%), *RAS* status (wild/mutant/unknown: 32/54/16 and 76/16/8%). Response rate was 8.6% in BBP group and 9.1% in non-BBP group (*p* = 1.00). Median PFS was 3.9 months in BBP group and 2.8 months in non-BBP group (HR [95%CI]: 0.79 [0.46–1.34], *p* = 0.373, adjusted HR: 0.87 [0.41–1.82], *p* = 0.707). Median overall survival was 8.5 months in BBP group and 5.4 months in non-BBP group (HR 0.66 [0.38–1.12], *p* = 0.125, adjusted HR 0.53 [0.27–1.07], *p* = 0.078).

**Conclusion:**

In mCRC patients who experienced early progression in first-line chemotherapy, second-line chemotherapy showed poor clinical outcomes regardless use of anti-angiogenic agents.

**Supplementary Information:**

The online version contains supplementary material available at 10.1186/s12885-021-08890-6.

## Background

For patients with unresectable metastatic colorectal cancer (mCRC), systemic chemotherapy is recognized as the standard treatment worldwide [1–3]. Cytotoxic doublet or triplet chemotherapy plus targeted agent, such as bevacizumab (BEV) or anti-epidermal growth factor receptor (EGFR) inhibitor, is recommended as the first-line chemotherapy [3]. After failure of chemotherapy with BEV as first-line treatment, two large observational studies suggested that BEV continuation beyond progression (BBP) might improve the prognosis [4, 5]. Since a randomized trial (ML18147) showed a survival benefit of BBP compared to chemotherapy alone in the second-line setting (Hazard ratio [HR], 0.81; 95% confidence interval [CI], 0.69–0.94; *p* = 0.0062) [6], it has been adopted as one of the standard treatment strategies in second-line chemotherapy for mCRC patients [3]. However, the ML18147 trial excluded mCRC patients whose progression-free survival (PFS) during first-line chemotherapy was less than 3 months, as well as those in whom progressive disease was observed later than 3 months after the last BEV administration, or those who were treated with first-line chemotherapy containing BEV for less than 3 months [6].

Antiangiogenic agents are generally considered less likely to induce drug-resistance than cytotoxic agents because antiangiogenic agents act mainly on endothelial cells rather than on tumor cells [7]. However, it is not known whether BBP would have clinical benefits for patients who experienced disease progression within 3 months during a first-line BEV-containing chemotherapy; this patient group may be intrinsically resistant to BEV.

Here, we conducted a multi-institutional retrospective study to evaluate the efficacy and safety of second-line chemotherapy with or without BEV/other anti-angiogenic agents after early disease progression within 3 months during first-line BEV-containing chemotherapy.

## Methods

### Patients

This multi-institutional retrospective study was conducted at 9 Japanese hospitals. The main selection criteria of the subjects were; histologically confirmed unresectable or recurrent mCRC, age > 18 years, PFS < 100 days (early disease progression) during first-line chemotherapy containing BEV in combination with doublet cytotoxic agents, fluoropyrimidine, and oxaliplatin or irinotecan, which was initiated from Apr 2007 to Dec 2016. Additionally, patients who were intolerant to the first-line chemotherapy containing BEV were excluded. This study was approved by all the participating institutional review boards. Because of the retrospective nature of this study, informed consent was not obtained from each patient.

### Treatments

Patients were divided into the two groups according to the second-line chemotherapy regimens with or without BEV/other anti-angiogenic agents, and were subsequently described as subjects in the BBP and non-BBP groups, respectively. In the BBP group, patients received cytotoxic agents with BEV at 2.5 mg/kg per week (5.0 mg/kg every 2 weeks or 7.5 mg/kg every 3 weeks). In the non-BBP group, patients received single or doublet cytotoxic agents with or without anti- EGFR antibody, or anti-EGFR antibody alone.

### Evaluations

Tumor responses were evaluated according to the Response Evaluation Criteria in Solid Tumors (RECIST) version 1.1. Toxicity was assessed using the Common Terminology Criteria for Adverse Events (CTCAE) version 4.0. PFS was defined as the time from the initiation of second-line chemotherapy to disease progression or death from any cause, and was censored at the last visit of patients surviving without documented disease progression. Overall survival (OS) was defined as time from the initiation of the second-line chemotherapy to death or censored at the last visit of surviving patients.

Survival curves for PFS and OS were estimated by the Kaplan-Meier method and confidence intervals (CI) were calculated based on the Greenwood formula. The confidence intervals of median survival time were calculated using the Brookmeyer-Crowley method. Hazard ratio (HR) and adjusted HR were obtained using Cox regression models with well-known prognostic factors (performance status [PS]), alkaline phosphatase (ALP), white blood cells (WBC), the number of metastatic organ sites [8], *RAS* status [9–11], sidedness [12–14] in multi-variate analysis. *P*-value < 0.05 as determined using the two-tailed test was considered to be significant. SAS version 9.4 was used for all statistical analysis.

## Results

### Patient characteristics

A total of 62 patients across 9 institutions were identified as the subjects of this study. According to the second-line chemotherapy regimens, 37 and 25 patents were classified into the BBP and non-BBP groups, respectively. The patients’ baseline characteristics are summarized in Table 1. In the BBP group, 33 patients (89.2%) were PS ≤1, 11 patients (32.4%) had *KRAS/RAS* wildtype, and 8 patients (21.6%) had peritoneal metastasis. In the non-BBP group, 14 patients (56.0%) were PS ≤1, and 19 patients (76.0%) had *KRAS/RAS* wild type, and 12 patients (48.0%) had peritoneum metastasis. There were no significant differences in age, sex, primary tumor location, disease status, number of metastatic organ sites or the first-line chemotherapy regimens between the two groups. Among the patients with measurable lesions, the best responses without confirmation in the first-line chemotherapy were stable disease in 6 patients (16.2%) and progressive disease in 29 (78.4%) of the BBP group and stable disease was seen in 6 (27.2%) patients and progressive disease was observed in 15 patients (68.2%) of the non-BBP group.

**Table 1:** Baseline characteristics of eligible patients

	BBP group (%) N = 37	Non-BBP group (%) *N* = 25	*P*-value
Sex			
Male	19 (51.4)	8 (32.0)	0.192
Female	18 (48.6)	17 (68.0)	
Age (years)			
Median (range)	59 (34–82)	57 (27–74)	0.503
ECOG PS			
0	12 (32.4)	7 (28.0)	0.010
1	21 (56.8)	7 (28.0)	
2≤	3 (8.1)	10 (40.0)	
Unknown	1 (2.7)	1 (4.0)	
RAS status			
KRAS/RAS wild type	11 (32.4)	19 (76.0)	0.001
KRAS/RAS mutant	20 (54.0)	4 (16.0)	
Unknown	6 (16.2)	2 (8.0)	
Primary location			
Right side^a^	16 (43.2)	10 (40.0)	1.000
Left side^b^	21 (56.8)	15 (60.0)	
Disease status			
Stage IV	24 (64.9)	21 (84.0)	0.147
Recurrence	13 (35.1)	4 (16.0)	
Number of metastatic organ sites			
1	13 (35.1)	5 (20.0)	0.259
2≤	24 (64.9)	20 (80.0)	
Metastatic sites			
Liver	32 (86.5)	18 (72.0)	0.198
Lung	14 (37.8)	9 (36.0)	1.000
Lymph node	13 (35.1)	12 (48.0)	0.429
Peritoneum	8 (21.6)	12 (48.0)	0.051
First-line chemotherapy			
Oxaliplatin-based regimen	31 (83.8)	24 (96.0)	0.225
Irinotecan-based regimen	6 (16.2)	1 (4.0)	

*Abbreviation*: *BBP* Bevacizumab continuation beyond progression

^a^: Appendix, caecum, ascending colon, hepatic flexure and transverse colon

^b^: Splenic flexure, descending colon, sigmoid colon and rectum

### Second-line regimens

The regimens in the second-line chemotherapy are shown in Table 2. All patients in the BBP group received doublet chemotherapy plus BEV, while 16 (64%) and 9 (36%) patients in the non-BBP group were treated with anti-EGFR antibody-containing regimens or cytotoxic agents alone.

**Table 2:** Second-line chemotherapy which eligible patients received and tumor response evaluated RECIST ver1.1

Regimen	BBP group (%)	Non-BBP group (%)
Oxaliplatin-based		
FOLFOX	0 (0)	1 (4.0)
FOLFOX+BEV	7 (18.9)	0 (0)
Irinotecan-based		
FOLFIRI	0 (0)	4 (16.0)
FOLFIRI+CET/PANI	0 (0)	8 (32.0)
Irinotecan	0 (0)	4 (16.0)
Irinotecan+CET/PANI	0 (0)	4 (16.0)
FOLFIRI+BEV	26 (70.3)	0 (0)
IRIS+BEV	2 (5.4)	0 (0)
XELIRI+BEV	1 (2.7)	0 (0)
FOLFIRI+RAM	1 (2.7)	0 (0)
EGFR antibody alone		
CET/PANI	0 (0)	4 (16.0)
Tumor response		
Best response	BBP group^a^ (%) *N* = 35	Non-BBP group^a^ (%) *N* = 22
Complete response	0 (0)	0 (0)
Partial response	3 (8.6)	2 (9.1)
Stable disease	15 (42.9)	6 (27.3)
Progressive disease	14 (40.0)	12 (54.5)
Not evaluated	3 (8.6)	2 (9.1)
Objective response rate	3 (8.6)	2 (9.1)
Disease control rate	18 (51.4)	8 (36.4)

*Abbreviations*: *BBP* Bevacizumab continuation beyond progression, *BEV* Bevacizumab, *FOLFOX* 5-FU and leucovorin, oxaliplatin, *FOLFIRI* 5-FU and leucovorin, irinotecan, *IRIS* Irinotecan and S-1, *XELIRI* capecitabine and irinotecan, *RAM* Ramucirumab, *CET* Cetuximab, *PANI* Panitumumab, *EGFP* Epidermal growth factor receptor

^a^Patients who had measurable lesions

### Efficacy

**Fig. 1:**
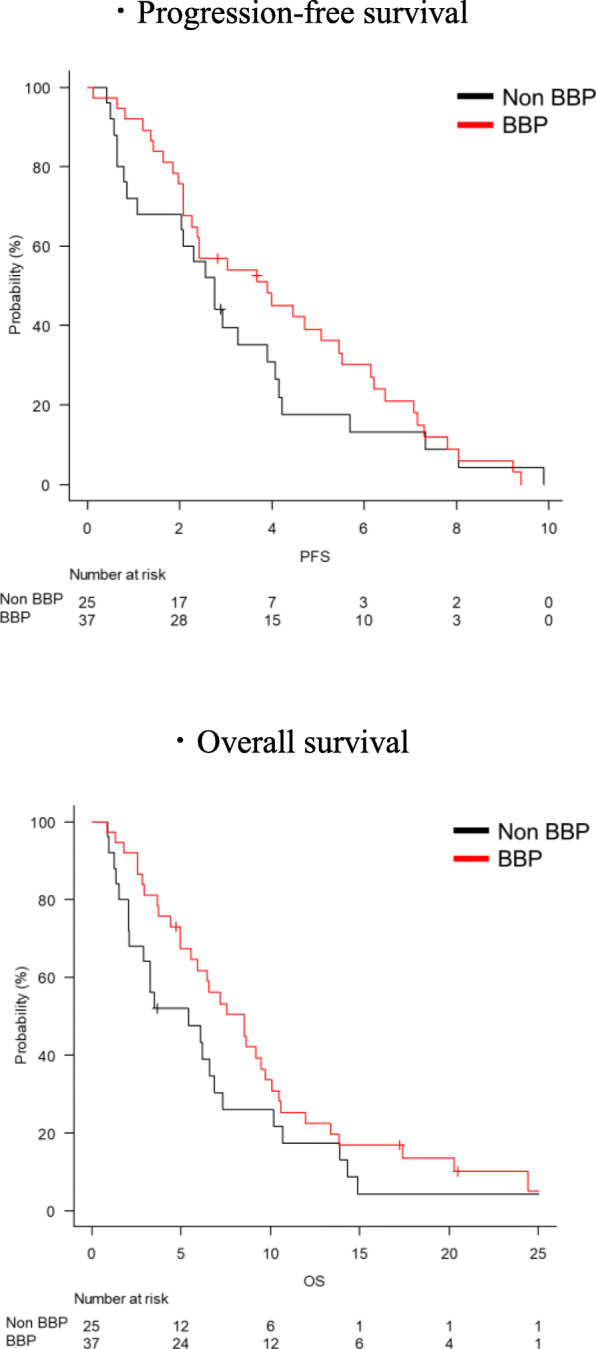
Kaplan-Meier survival curves of progression-free survival and overall survival in the BBP group (*n* = 37) and in the non-BBP group (*n* = 25)

Two patients in the BBP group and three patients in the non-BBP group did not have measurable lesions. Among the patients with measurable lesions, the objective response and disease control rates were 8.6% (*n* = 3) and 51.4% in the BBP group and 9.1% (*n* = 2) and 36.4% in the non-BBP group (Table 2). With the median follow-up time was 6.2 months, median PFS was 3.9 months (95%CI: 2.3–5.5) in the BBP group, and 2.8 months (95%CI: 1.1–4.0) in the non-BBP group (HR [95%CI]: 0.79 [0.46–1.34], *p* = 0.373, adjusted HR [95%CI]: 0.87 [0.41–1.82], *p* = 0.707, Fig. 1, Table S1). Median OS was 8.5 months (95%CI: 4.9–9.7) in the BBP group, and 5.4 months (95%CI: 2.1–6.9) in the non-BBP group (HR [95%CI]: 0.66 [0.38–1.12], *p* = 0.125, adjusted HR [95%CI]: 0.53 [0.27–1.07], *p* = 0.078, Fig. 1, Table S1). PFS of three patients in the BBP group who had a partial response in the second line chemotherapy were as short as 3.0, 4.5, 9.3 months and OS were 6.4 9.5 20.5 months. In contrast, PFS of two responders in the non-BBP group were 8.0 and 9.9 months and OS were 13.9 months and 14.9 months. With regard to the patients who received combination chemotherapy with anti-EGFR antibody in the non-BBP group, the objective response and disease control rates were 22.2% (2/9 patients) and 44.4% (4/9 patients), while median PFS and OS were 3.3 months (95%CI: 0.7–7.3) and 4.9 months (95%CI: 1.2–13.9).

### Treatment after second-line chemotherapy

All subsequent treatments after the second-line chemotherapy in both groups are shown in Table 3. In the BBP group, 21 patients (56.8%) had subsequent treatments after the second-line chemotherapy: irinotecan-based chemotherapy in 17 patients (45.9%) and TAS-102 in 15 patients (40.5%), while in the non-BBP group, only 8 patients (32.0%) received subsequent treatments: regorafenib in 2 patients (8.0%), anti-EGFR antibody monotherapy in 2 patients (8.0%). irinotecan-based chemotherapy in 2 patients (8.0%).

**Table 3:** Treatment after the second-line chemotherapy

Treatment	BBP group (%)	Non-BBP group (%)
No	16 (43.2)	17 (68.0)
Yes (overlapping)	21 (56.8)	8 (32.0)
TAS-102	15 (40.5)	6 (26.1)
REGO	14 (37.8)	13 (50.0)
CET/PANI	3 (8.1)	2 (8.7)
Oxaliplatin-based	2 (5.4)	2 (8.7)
Irinotecan-based	17 (45.9)	8 (34.8)
Hepatic arterial infusion	4 (10.8)	4 (11.1)
Others	4 (10.8)	4 (16.0)

*Abbreviations*: *BBP* Bevacizumab continuation beyond progression, *REGO* Regorafenib, *CET* Cetuximab, *PANI* Panitumumab

### Safety

Adverse events during second-line chemotherapy are summarized in Table 4. The most common grade 3 or 4 adverse event was neutropenia in both the BBP and the non-BBP groups. In the BBP group, one patient (2.7%) had grade 3 febrile neutropenia. In the non-BBP group, 4 patients (16.0%) had grade 3–4 anemia and 2 patients (8.0%) had grade 3–4 anorexia. In the BBP group, hemorrhage, hypertension, proteinuria, embolism, and gastrointestinal perforation were rare.

**Table 4:** Grade 3 ≤ adverse events during the second-line chemotherapy

Adverse event	BBP group (%)	Non-BBP group (%)
Hematological		
Neutropenia	6 (16.2)	6 (24.0)
Anemia	1 (2.7)	4 (16.0)
Thrombocytopenia	1 (2.7)	1 (4.0)
Non-hematological		
Fatigue	0 (0)	1 (4.0)
Decreased appetite	2 (5.4)	2 (8.0)
Diarrhea	2 (5.4)	0 (0)
Hypertension	1 (2.7)	0 (0)
Proteinuria	1 (2.7)	0 (0)
Gastrointestinal perforation	1 (2.7)	1 (4.0)
Bleeding	1 (2.7)	1 (4.0)
Venous thromboembolic	1 (2.7)	0 (0)
Cerebral infarction	1 (2.7)	0 (0)
Ileus	1 (2.7)	1 (4.0)
Hepatic infection	0 (0)	1 (4.0).
Febrile neutropenia	1 (2.7)	0 (0)

## Discussion

BBP has been recognized as a standard second-line treatment following the results of the ML18147 trial, in which median PFS and OS were 5.7 months and 11.2 months, respectively [6]. On the other hand, median PFS and OS when anti-EGFR antibody plus chemotherapy in the second-line treatment were 4.0–6.0 months and 10.7–16.2 months, respectively [15–17]. In this study, median PFS and OS were not significantly different between the BBP and non-BBP groups. Indeed, compared with the reported results of the randomized controlled trials of BBP and anti-EGFR antibody, the subjects in our study showed poor clinical outcomes regardless of the treatment strategy, BBP or non-BBP. In a randomized phase III trial for mCRC, the proportion of patients with early disease progression during first-line chemotherapy was only 3–4% [18]; this is similar to the incidence of early progression (4.1%) in patients allocated to the BEV-containing arm in the TRICOLORE trial [19]. Although the number of patients with early disease progression during BEV-containing first-line chemotherapy is small, we believe it is important to develop effective therapies for these patients.

There were substantial differences in patients’ backgrounds between the two groups in the present study. The proportion of patients with PS ≥2 was higher in the non-BBP group (40.0%) than in the BBP group (8.1%). All patients in the BBP group received doublet plus BEV, while only 8 patients (32.0%) in the non-BBP group received doublet plus a targeted agent. The proportion of patients with *RAS* wild type was higher in the non-BBP group (76.0%) than in the BBP group (32.4%), and 16 patients (64%) in the non-BBP group received chemotherapy containing anti-EGFR antibody, which is recommended by current guidelines [1–3]. These differences in patients’ conditions seem to have influenced the selection of chemotherapy regimens.

In this study, the adjusted HR for OS when BBP and non-BBP groups were compared was 0.66, while the adjusted HR for PFS was 0.87. However, in addition to the differences in patient’s background and chemotherapy regimens, there was a substantial difference in the proportion of patients receiving subsequent chemotherapy. Given these issues, it was challenging to compare efficacy between the two groups, especially for OS, even after adjusting for patient backgrounds with multivariate analysis. Limited to the patients who received standard treatment strategy both in the first- and second line chemotherapy, all 37 patients in the BBP group and 12 of 25 patients in the non-BBP group received combination chemotherapy of cytotoxic agents and molecular target agents. Among these 49 patients, there were some differences in PS and *KRAS/RAS* status: proportions of PS 0–1? 89.258.3%, and those of *KRAS/RAS* wild type 32.4 and 100% in BBP and non BBP groups. These poor condition of these 12 patients of the non-BBP group might result in short mPFS and mOS as 3.3 and 4.9 months. Thus, it is still difficult to compared between BBP and non-BBP even along with the standard treatment strategy. Therefore, it remains unclear whether BBP or non-BBP is the preferable treatment option for patients with early disease progression during first-line chemotherapy containing BEV.

As for the response to chemotherapy, the response rate (8.6%) in the BBP group of this study was very similar to that (5.4%) of the BBP group in the ML18147 trial; however, the disease control rate (51.4%) in our study was lower than that (68%) in ML18147 [6]. Conversely, the response rate in the non-BBP group in our study was slightly higher (9.1%) than in the BBP group, but the disease control rate lower at 36.4%. Based on the survival analysis, we think it is reasonable to speculate that many of the subjects in this study would have had tumors that were resistant to the agents used in second-line chemotherapy, regardless of whether they were in the BBP or non-BBP group. However, survival analysis results by best response in this study showed that better response in the second-line chemotherapy might lead to better clinical outcomes. It is considered that response/disease control in the second-line chemotherapy may be important to improve clinical outcomes of patients even with early disease progression in the first-line chemotherapy.

Biomarker analysis of the CALGB/SWOG 80405 trial showed that low baseline levels of serum VEGF-D were specifically predictive of longer PFS in patients who received FOLFOX plus BEV than in patients who received FOLFOX plus cetuximab as the first-line chemotherapy [20]. BEV suppresses tumor angiogenesis mainly by inhibiting the binding of VEGF-A to VEGFR-1/2. However, VEGF-D also binds to VEGFR-2 and promotes tumor angiogenesis, and therefore BEV efficacy may be poor in patients with high levels of serum VEGF-D. In addition to VEGF-D, biomarker studies suggest that VEGF-A [21] and PIGF [21, 22] might be associated with the efficacy of anti-angiogenic therapy. Therefore, we speculate that elevated activity of angiogenic pathway might be responsible for BEV resistance of some subjects in this study.

RAM and AFL are also anti-angiogenic agents that inhibit the VEGF pathway by mechanisms different from BEV [23, 24]. RAM is a fully human IgG-1 monoclonal antibody, and exerts its antitumor activity by binding to the VEGFR-2 extracellular domain [24, 25]. Subgroup analysis of the RAISE study showed that RAM was efficacious in patients with high levels of VEGF-D [26]. In addition, subgroup analysis of patients with first-line TTP < 6 months in the RAISE trial showed that median OS was 10.4 months versus 8.0 months (HR [95%CI]: 0.86 [0.64–1.13], *p* = 0.2759), and median PFS was 5.2 months versus 2.9 months (HR [95%CI]: 0.68[0.52–0.89], *p* = 0.0042) [27]. Actually in the present study, one patient who received FOLFIRI plus ramucirumab showed very favorable PFS and OS (Table S1). We suggest that future clinical researches should evaluate the benefits of switching to RAM for patients with high levels of VEGF-D who experience early progression during first-line chemotherapy containing BEV. AFL is a recombinant fusion protein that prevents VEGF-A, VEGF-B, and PIGF-1 from binding to their cognate receptors [23]. Subgroup analysis of patients with first-line TTP < 3 months in the VELOUR trial showed that median OS was 11.9 months in the AFL plus FOLFIRI group versus 9.8 months in placebo plus FOLFIRI (HR, 0.63), while median PFS was 7.0 months versus 3.9 months (HR, 0.55) [28]. These data, which are based on randomized trials, suggest that switching to other anti-angiogenic agents during second-line chemotherapy may be a treatment option for patients who had early disease progression during first-line BEV-containing chemotherapy. However, in the VELOUR trial, only 30.4% of patients received BEV as part of the first-line chemotherapy [28], whereas the number of patients who had TTP < 3 months during first-line chemotherapy and who received BEV containing chemotherapy was not clear. Further studies will be needed to test the validity of such an approach.

As for the non-BBP group in this study, it is very difficult to compare their response and disease control rates with those previously reported, because the non-BBP group contained various kinds of chemotherapy regimens. The majority of the patients who received combination chemotherapy with anti-EGFR antibody had a poor prognosis. Moreover, in a previous retrospective analysis, the efficacy of anti-EGFR antibody-containing treatments as second- or third-line chemotherapy was poorer in patients who previously received BEV than in patients without prior BEV [29]. It has been reported that patients who received a BEV-containing regimen had higher levels of VEGF-A than the patients without prior BEV [29]. Furthermore, experiments with CRC cell lines suggest that high levels of VEGF-A induce VEGFR-2- and STAT-3-dependent resistance to cetuximab [29]. Another preclinical study suggested that BEV-resistant CRC cells had higher levels of VEGF due to upregulation of the hypoxia induced factor (HIF)-VEGF signaling pathway [30]. We note that HIFs can also activate EGFR and the associated downstream signal transduction pathways [31]. These molecular mechanisms might underlie the poor efficacy of anti-EGFR antibody-containing regimens that we observed in this study. We suggest that the identification of biomarkers that aid selection of anti-EGFR antibody-containing chemotherapy for patients who experience early progression during first-line chemotherapy containing BEV should be prioritized.

This study has some limitations. First, it is a small retrospective study, leading to some bias in patients’ characteristics such as PS and selection of chemotherapy based on *RAS* mutational status. These imbalances might affect the survival outcomes in this study, which might not be adjusted even by multivariate analysis. Additionally, we retrospectively collected data during 10 years because of small population, and clinical practice such as mitigations, co-medications, monitoring and treatment of side effects and diagnostic methods changed during these 10 years. These factors might have some impact on the results of this study. Secondly, in some patients, we could not collect information of *BRAF* mutational status (unknown: *n* = 46); these genes are well-known prognostic factors in colorectal cancer [32–34]. Finally, we could not perform antiangiogenic biomarker analysis of molecules such as VEGF-D and A [20, 26] due to retrospective study design. As a consequence of these limitations, the question whether BEV continuation beyond progressions or not is more favorable cannot be answered by the results of this study. However, to the best of our knowledge, this is the first report that clarifies the efficacy and safety of BBP or non-BBP strategies as second-line chemotherapy for mCRC patients with early disease progression during first-line BEV-containing chemotherapy.

## Conclusions

For mCRC patients with early disease progression in the first-line setting, the efficacy of second-line chemotherapy is modest, regardless of whether BBP or other strategies have been employed. The optimal treatment for these mCRC patients, BBP or other strategies could not be clarified. To improve clinical outcomes of these patients, new treatment strategies are warranted.

## Supplementary Information

**Additional file 1: Table 1S.** Progression-free survival and overall survival by second-line chemotherapy regimen.

## Data Availability

The datasets generated during this study are not publicly available due to ethical restrictions, but these are available from the corresponding author on reasonable request.

## Abbreviations

AFL: Aflibercept
ALP: Alkaline phosphatase
BEV: Bevacizumab
BBP: BEV continuation beyond progression
CI: Confidence interval
CTCAE: Common Terminology Criteria for Adverse Events
EGFR: Epidermal growth factor receptor
HIF: Hypoxia induced factor
HR: Hazard ratio
mCRC: Metastatic colorectal cancer
OS: Overall survival
PFS: Progression-free survival
PS: Performance status
RAM: Ramucirumab
RECIST: Response Evaluation Criteria in Solid Tumors
WBC: White blood cells
