# Supplementary material for: Second-line chemotherapy after early disease progression during first-line chemotherapy containing bevacizumab for patients with metastatic colorectal cancer
Source: BMC Cancer. 2021 Oct 29;21:1159. doi: 10.1186/s12885-021-08890-6 (PMC8555183; doi:10.1186/s12885-021-08890-6)
Supplement: Supplementary file 1 — Additional file 1: Table 1S. Progression-free survival and overall survival by second-line chemotherapy regimen. [file 12885_2021_8890_MOESM1_ESM.docx]

**Table 1S. Progression-free survival and overall survival by second-line chemotherapy regimen**

| Regimen | Number of patients | mPFS  (month, 95%CI) | mOS  (month, 95%CI) |
| --- | --- | --- | --- |
| BBP group | | | |
| FOLFIRI+BEV | 26 | 3.1 (2.0-5.1) | 8.6 (3.7-9.5) |
| FOLFOX+BEV | 7 | 4.7 (1.8-7.3) | 10.1 (4.9-10.6) |
| IRIS+BEV | 2 | 4.4 (2.4-NA) | 6.6 (4.4 NA) |
| XELIRI+BEV | 1 | 7.1 (NA) | 17.4 (NA) |
| FOLFIRI+RAM | 1 | 9.4 (NA) | 20.5 (NA) |
| Non BBP group | | | |
| Cytotoxic agent + EGFR antibody |  |  |  |
| FOLFIRI+CET/PANI | 8 | 2.7 (0.7-7.3) | 3.4 (1.2-13.9) |
| Irinotecan+CET/PANI | 4 | 3.7 (0.7-NA) | 8.4 (0.9-NA) |
| EGFR antibody alone |  |  |  |
| CET/PANI | 4 | 0.8 (0.5-NA) | 1.8 (1.0-NA) |
| Cytotoxic Agent alone |  |  |  |
| FOLFIRI | 4 | 1.8 (0.4-NA) | 6.4 (2.1-NA) |
| Irinotecan | 4 | 3.3 (2.1-NA) | 6.1 (3.3-NA) |
| FOLFOX | 1 | 2.9 (NA) | 6.6 (NA) |

Abbreviations: PFS, progression-free survival; OS, overall survival; BEV, bevacizumab; FOLFOX, 5-FU and leucovorin, oxaliplatin; FOLFIRI, 5-FU and leucovorin, irinotecan; IRIS, irinotecan and S-1; XELIRI, capecitabine and irinotecan; RAM, ramucirumab; CET, cetuximab; PANI, panitumumab; EGFP, epidermal growth factor receptor.
